# Leptin may enhance hepatic insulin sensitivity in children and women born small for gestational age

**DOI:** 10.1530/EC-12-0071

**Published:** 2013-01-25

**Authors:** Anna Kistner, Mireille Vanpée, Kerstin Hall

**Affiliations:** 1 Department of Molecular Medicine and Surgery Karolinska Institutet SE-171 76, Stockholm Sweden; 2 Department of Women and Child Health Karolinska Institutet SE-171 76, StockholmSweden

**Keywords:** preterm children, SGA, BMI, insulin resistance, leptin, IGFBP1

## Abstract

**Objective:**

Children born small for gestational age (SGA) are at risk for developing type 2 diabetes. Lipodystrophy leads to early type 2 diabetes and leptin reverses the metabolic consequences of the disease. Low IGF-binding protein 1 (IGFBP1) can predict the development of type 2 diabetes. The aim of this study was to determine leptin, insulin, and IGFBP1 in children and adult women born preterm or SGA to evaluate the role of leptin as a compensatory mechanism in insulin resistance development.

**Methods:**

Seventy-six children (8.5–10 years, 41 girls and 35 boys) and 45 women (23–30 years) were studied. The children comprised subjects born appropriate for gestational age (<30 gestational weeks) (*n*=22), born SGA at term (*n*=23), and full-term normal-weight controls (*n*=31). Among the women, the corresponding figures were, *n*=10, *n*=18, and *n*=17 respectively. Fasting levels of IGFBP1, leptin, insulin, and IGF1 were determined and total adiponectin only in women.

**Results:**

In girls and women, term SGA subjects had higher leptin levels in relation to BMI SDS (*P*=0.042 and *P*=0.03 respectively). More than half of IGFBP1 variability was explained by leptin and insulin in children. In term SGA women, IGFBP1 level was lower compared with controls (*P*=0.012) and the regression line of IGFBP1 on insulin was suppressed below −1 s.d. of a reference material.

**Conclusion:**

Leptin levels were elevated in term SGA girls and women, in particular in adult women, but not found in preterm girls and women. IGFBP1 was lower in term SGA women. In children, leptin and insulin were strong suppressors of IGFBP1. We speculate that higher leptin levels could be a protective event to enhance hepatic insulin sensitivity.

## Introduction

Low birth weight (LBW) is a consequence of either intrauterine growth restriction (IUGR) resulting in infants born small for gestational age (SGA) or due to preterm interruption of gestation or a combination of these two. LBW (defined as birth weight (BW) below 2500 g) has been proposed to lead to insulin resistance and type 2 diabetes (1, 2, 3, 4). In studies of individuals born SGA at term (defined as BW SDS below −2 SDS), signs of insulin resistance have been detected in children (5) and at young adulthood (6). In our group of children born SGA at term (defined as BW SDS below −2 SDS), we have recently found higher insulin levels compared with the controls when adjusted for BMI, indicating early signs of a peripheral insulin resistance (7).

In some studies, under-nutrition *in utero* has been associated with increased catch-up growth in childhood, increased relative percent body fat, and obesity (8, 9, 10, 11). It is known that the development of type 2 diabetes is associated with obesity. Increased body fat percentage in humans has been shown to be correlated with higher leptin levels (12). Leptin is a peptide hormone produced and secreted from the white adipose tissue (13). In a population of relatively lean adult Chinese men and women, increased leptin levels were associated with insulin resistance and prediabetes (14), although the causal relation was not revealed. In lipodystrophy, where individuals lack leptin and suffer from severe insulin resistance, leptin therapy has been shown to be effective in treating the metabolic effects of the disease (15). Leptin receptors are found in human liver cells and in many organs of the body (16). In SGA children with signs of insulin resistance, results are conflicting concerning leptin levels (5, 17, 18, 19) but elevated levels have been observed in prepubertal SGA children with catch-up growth (19).

IGF-binding protein-1 (IGFBP1) modulates the effect of IGF1 at target tissue. IGFBP1 levels are depending on insulin action on the liver and can be used as a surrogate marker for hepatic glucose production (20). Its expression in hepatocytes is suppressed by insulin. In prospective epidemiological studies on men and women with normal oral glucose tolerance test (OGTT), low IGFBP1 predicts the development of type 2 diabetes within a decade (21, 22). In young adult women born SGA at term, we have previously shown decreased levels of IGFBP1 compared with controls (23).

The aim of this study was to determine leptin, insulin, and IGFBP1 levels in a group of children and in adult women born either with LBW due to preterm birth or born SGA at term in order to evaluate the possible role of leptin as a compensatory mechanism in insulin resistance development. Our hypothesis is that leptin may enhance hepatic insulin sensitivity and exert a protective property.

## Materials and methods

### Subjects

The characterizations and the modes of selection for the children (7) and the adult women (23) were previously described in detail. In brief, of 257 children consequently born at the Karolinska University Hospital between the years 1990 and 1993 and invited to take part in a follow-up study, 105 excepted to participate and blood values were obtained in 76 subjects (7). For women, a total of 77 female infants born preterm before gestational week 32 or born term SGA in the Stockholm area between the years 1967 and 1973 were contacted to take part in this study. At birth, a large portion of the mothers in the term SGA group had participated in a study because of giving birth to a SGA infant. Thirty-two women born preterm or term SGA agreed to participate in this study and 17 age-matched women controls were included. In an initial cohort of 50 adult women, blood values were obtained in 45 subjects (23). The subjects were divided into three groups according to preterm birth with BW SDS>−2 for gestational age (GA) (preterm appropriate for gestational age (AGA)), full-term birth between 37 and 42 GA but BW SDS<−2 (term SGA), or full-term birth and AGA (control). Children and women born preterm but also SGA were eliminated from the initial cohorts (two boys, four girls, and five women).

The 76 children included were 22 preterm AGA born <30 weeks GA (13 boys and 9 girls), 23 term SGA children (10 boys and 13 girls), and 31 controls (12 boys and 19 girls) (Table 1). Birth characteristics for the participating children did not differ from the nonparticipating subjects in the initial cohorts (22/32 preterm AGA, 23/30 SGA, and 31/36 control). Mean BW in the participating vs nonparticipating subjects (mean±s.d., g) was 902±183 vs 977±222 (*P*=0.36, ANOVAs, *t*-test), 2477±290 vs 2356±461 (*P*=0.41), and 3556±502 vs 3179±325 (*P*=0.12) in the preterm AGA, term SGA, and controls respectively. At the time of investigation, all 76 children were at Tanner stage <2 according to breast/genital.

The children were divided into boys and girls because of the large difference in leptin levels between sexes. This subdivision of the children also entitled comparisons between girls and women, as we did not have results from a group of 25-year-old men.

The women group comprised 45 subjects aged between 23 and 30 years (mean 26±2 years): ten preterm AGA born <32 weeks GA, 18 term SGA, and 17 controls. Term SGA girls had higher BW SDS (*P*<0.001) compared with term SGA women (Table 1). GA in the preterm AGA and control was lower in girls than in women (*P*<0.001 and *P*<0.01 respectively) (Table 1). The maternal age in the preterm AGA was higher in girls than in women (*P*<0.05) (Table 1). The maternal pre-pregnancy weight in the preterm AGA women was greater compared with term SGA women (*P*=0.039) (Table 1). Maternal pregnancy weight gain was lower in preterm AGA boys and girls and Term SGA girls compared to controls (*P*<0.001, *P*=0.043 and *P*=0.044 respectively, Table 1).

Four mothers in the children's group developed preeclampsia (two preterm AGA boys, one preterm AGA girl, and one term SGA boy) during pregnancy and one mother (a term SGA boy) developed gestational diabetes. Furthermore, four boys (one preterm AGA and three controls) had a maternal/paternal history for type 1 or 2 diabetes. In women, one mother developed preeclampsia (term SGA) during pregnancy. We do not have information concerning gestational diabetes in the women but the heredity for type 2 diabetes was ∼20% in each separate group and did not differ between groups. Maternal history for smoking during pregnancy in the children's groups was ∼20% in each separate group, whereas in the women's groups it was 20% in the preterm AGA, 61% in term SGA compared with 23.5% in controls (*P*=0.030, Pearson's *χ* ^2^ test). The placenta weight was (median (range)) (missing values) 410 (220–750) g (2), 405 (220–740) g (1), and 610 (430–760) g (7) (*P*=0.004, ANOVA test) in the preterm AGA, term SGA, and control women's groups respectively. We do not have information pertaining placenta weight in the children.

There were no significant differences with regard to smoking (∼40% of the women in each group smoked or smoked occasionally) or alcohol habits in the participating women groups. The contraceptives used were low-dose tablets (<50 μg ethinyl estradiol) and the frequency of use was 40, 22, and 53% in the preterm AGA, term SGA, and control groups respectively (*P*=0.17, Pearson's *χ* ^2^ test).

Two outliers were present. One woman in the control group with a high leptin level in relation to BMI in combination with elevated triglycerides was excluded from correlations with leptin. The other outlier excluded from mean IGFBP1 calculations was a term SGA woman with very high IGFBP1 (121 μg/l) accompanied by increased inflammation markers (neopterin 22.6 μg/l and vcam 595 pmol/l) suggesting a possible ongoing inflammation, which increases IGFBP1.

### Methods

#### Data collection

GA for the children was determined by ultrasound in early pregnancy whereas in women GA was estimated according to the first day of the last menstrual period and by evaluation of the maturity stage at birth by a neonatologist. The introduction of ultrasound to estimate GA must be regarded as a more precise method compared to earlier estimations. SGA was defined as a BW <−2 s.d. (i.e. below the sex-specific 2.5th centile for GA) according to Swedish reference data for normal fetal growth (24). SDS are based on the Swedish reference curve at birth (24) and at the time of the study (25) (Tables 1 and 2). In children, target height (Table 2) was calculated according to parental height ((father's height+mother's height)/2+6.5 (boys)−6.5 cm (girls)) cm and target height SDS according to the Swedish reference material in adult age (25).

Neonatal characteristics were collected from the Swedish Birth Records as well as maternal characteristics related to the pregnancy. Information pertaining to maternal age, smoking during pregnancy, and medical history was obtained at the time of the study. Paternal and maternal anthropometric characteristics were obtained by the written/oral questions asked before or at the time of the follow-up study and hospital visit.

The same research nurse preformed all anthropometric measurements and blood samplings in the children and the same physician for the women subjects. The same physician performed 90% of the physical examinations in children and another physician performed all the physical examinations in the adult women. At the time of the study, all children and women were in good health. The Karolinska University Hospital Ethical Committee approved the study. Written consent was also obtained from the parents of the children, the children, and the adult women participating in the study.

#### Fasting blood values and homeostasis model assessment insulin resistance index

Blood samples were drawn in the morning after an overnight fast. Serum leptin was analyzed with a human leptin RIA using HL-81K (Linco Research, Inc., St. Charles, MO, USA), where ^125^I activity is measured by gamma counter. The sensitivity of detection was 0.5 μg/l. The intra-assay coefficient of variation (CV) for the leptin analysis was 5%. The interassay CV was 4.5%.

In-house RIA was used for IGFBP1 with individual serum samples in the same assay. The RIA for IGFBP1 was performed according to Povoa *et al*. (1984), the sensitivity of the RIA was 3 μg/l with intra- and interassays CV of 3 and 10% respectively (21, 26). Blood glucose readings were performed and documented by the nursing staff using Hemocue Glucose 201+ (Hemocue AB, Ängelholm, Sweden) (27). In children, commercial kits were used for S-insulin by ELISA (Dako, Glostrup, Denmark). The detection limit was 3 pmol/l. The intra- and interassay CV was 6.7 and 7.5% respectively. In adult women, insulin was determined by an in-house RIA, using guinea pig antiserum 22 with a detection limit of 56 pmol/l and the intra- and interassay CV was 5 and 10% respectively. For comparison between subjects at 9 years and adult women, insulin values in women were corrected by a factor (individual results divided by 2) to achieve equivalent and comparable insulin levels (Table 3). The mean±s.d. ratio between insulin determined by Daco and in-house assay was 0.50±0.12, and therefore, the in-house value was approximately twofold higher. Homeostasis model assessment insulin resistance index (HOMA-IR) in children was calculated from the formula (fasting glucose×fasting insulin/22.5) (Table 3). In-house RIA was also used for IGF1 with individual serum samples in the same assay. IGF1 was measured after ethanol extraction and cryoprecipitation and using des(1–3) IGF1 as a ligand (28). The detection limit was 8 μg/l and the intra- and interassay CV were 4 and 11% respectively. Human adiponectin was analyzed by RIA using HADP-61HK (Linco Research, Inc.). The sensitivity of the adiponectin analysis was 1 μg/l. The intra- and interassay CV were 4 and 8.5% respectively.

### Statistical analysis

Anthropometrical data in boys, girls, and women are presented as median (range). Normal distribution was achieved by log-transformation of serum leptin, insulin, HOMA-IR, IGFBP1, IGF1, and adiponectin values and are presented as geometrical mean ±95% CIs.

The comparison between three independent groups was assessed by ANOVA (parametric ANOVA) test, followed by *post hoc* Fisher's test for comparisons between the separate groups. The comparison between the two age-groups girls and women was assessed by *t*-test, and in women, analysis of covariance (ANCOVA) was performed with the variable leptin where adjustments for BMI SDS were made between groups. Pearson's *χ* ^2^ test was used to compare numerical values in children or in women or differences between the two age-groups girls and women relating to maternal characteristics and heredity. Forward stepwise multiple regression analyses were also performed with weight SDS, height SDS, leptin, and IGFBP1 as dependent variables. Co-linearity was tested for BMI SDS and catch-up height growth. In Fig. 2 (leptin vs BMI SDS), the deviation from the regression line in the SGA group was obtained by calculating predicted leptin value according to the combined regression line in control and preterm girls (^2^log *y*=2.0505+1.1179*x) (Fig. 2a) or in women to the regression line in control women (^2^log *y*=3.1343+0.2382*x) (Fig. 2b), where *x* is BMI SDS. Mean deviation from the regression line was then calculated by subtracting predicted leptin from estimated leptin. A *P* value of <0.05 was considered significant and tendency to significance was used in the *P* range 0.1 to >0.05. The statistical analyses were performed using Statistical Stat Soft, version 10 (Tulsa, OK, USA).

## Results

### Anthropometric data

#### Weight at follow-up and catch-up from birth to follow-up

Growth variables for all study subjects at follow-up are shown in Table 2. Term SGA girls were thinner with lower weight SDS and BMI SDS than control girls (Table 2). In women, term SGA women were thinner compared with controls but no differences in BMI SDS were found between the groups.

#### Height at follow-up and catch-up from birth to follow-up

The majority (74%) of children had reached their target height SDS ±1 s.d. At the onset of puberty, target height alone explained 28% (*P*<0.001) of the variability of height SDS and increased to 34% after addition of birth length SDS. Preterm AGA boys and girls were younger and shorter compared with controls at follow-up (Table 2). Preterm AGA boys and girls and term SGA boys had lower height SDS than controls at follow-up (Table 2). Term SGA women were also shorter than their controls.

**Figure 1:**
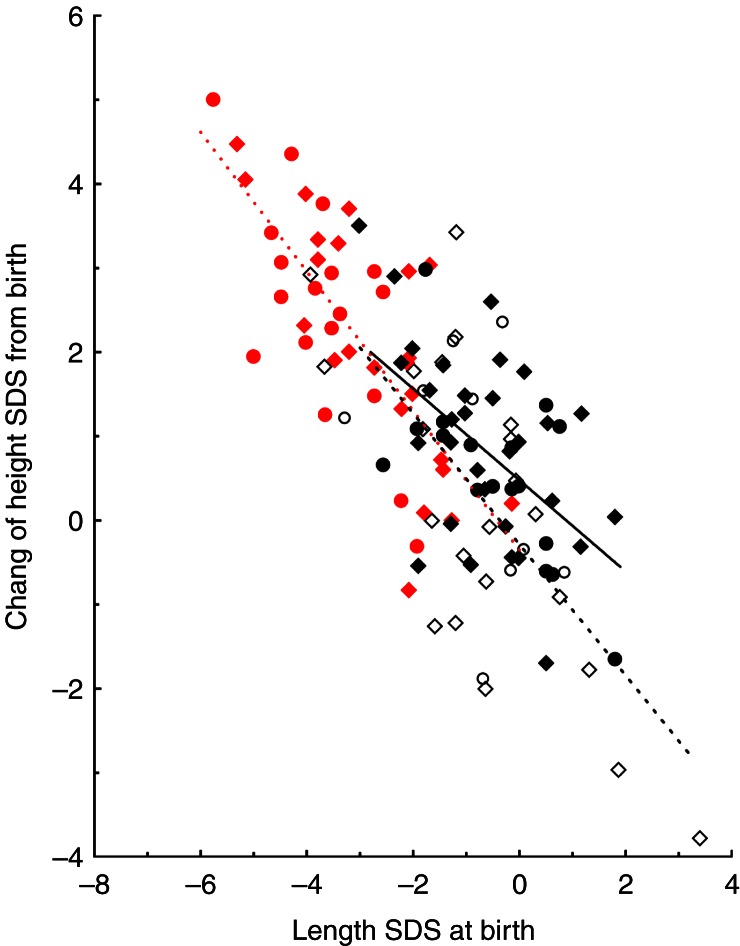
Catch-up height from birth to current age in relation to birth length SDS in children and women. Comparison between preterm AGA: (open diamond, children and open circle, women), term SGA: (closed red diamond, children and closed red circle, women), and control: (closed black diamond, children and closed black circle, women). The dotted black line represents all preterm AGA subjects (*y*=−0.2839−0.7792*x; *r*=−0.67, *P*=0.001), the dotted red line represents the term SGA subjects (*y*=−0.35−0.8276*x; *r*=−0.74, *P*<0.001), and the black solid line represents all controls (*y*=0.4798−0.5432*x; *r*=−0.56, *P*<0.001). There was no significant difference between children and women concerning these regression lines, but the line of control children crossed zero at +0.58 s.d. (*P*=0.010). Preterm AGA children with >1 s.d. decrease were born at 25 weeks or earlier.

In the three term SGA groups (boys, girls, and women), the median increase or catch-up of height SDS from birth to current age was ∼2 s.d. (Fig. 1 and Table 2). Height SDS catch-up growth was inversely correlated with birth length SDS with no significant difference in the regression lines found between children and women or between boys and girls (Fig. 1).

### Leptin

#### Leptin levels in children

**Figure 2:**
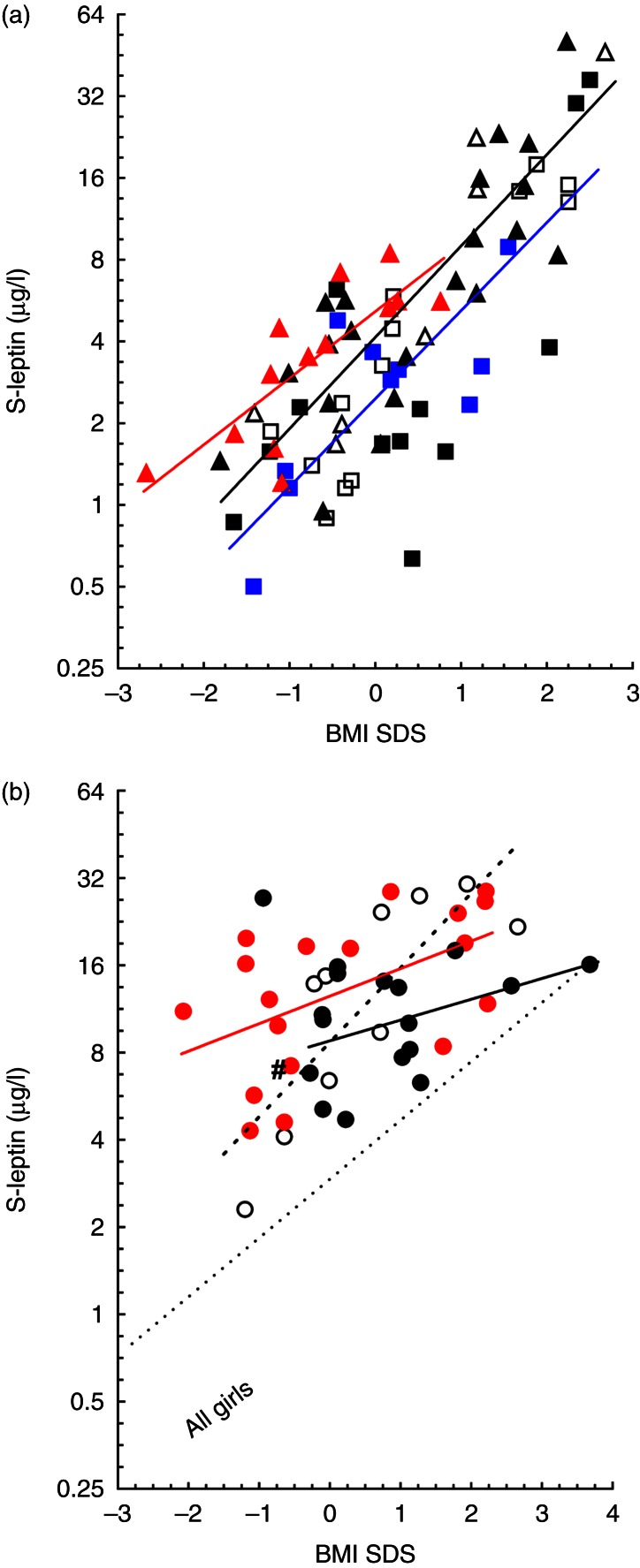
(a) Leptin levels (μg/l) in relation to BMI SDS in children. Comparison between girls and boys. Preterm AGA (open triangle, girls and open square, boys), term SGA: (closed red triangle, girls and closed blue square, boys), and control: (closed black triangle, girls and closed black square, boys). The red solid line represents term SGA girls (^2^log *y*=2.3663+0.8155*x, *r*=0.80, *P*<0.001) and the solid black line represents all other girls (^2^log *y*=2.0505+1.1179*x, *r*=0.85, *P*<0.001). The regression line in SGA girls was above that in the other girls (*P*=0.042). In boys, the regression lines did not differ between groups, the line in all boys (*n*=35) (^2^log *y*=1.299+1.0778*x, *r*=0.78, *P*<0.001) is shown in solid blue. (b) The relationship between leptin and BMI SDS in women. The different symbols of women are preterm AGA, open circle, term SGA, closed circle, and control, closed circle. The dotted line represents the preterm AGA group (^2^log *y*=3.1168+0.8538*x; *r*=0.81; *P*=0.005). The red solid line represents the term SGA group (^2^log *y*=3.6467+0.3154*x, *r*=0.52, *P*=0.028). The solid black line (^2^log *y*=3.1343+0.2382*x; *r*=0.42; *P*=0.106) represents the controls with one outlier excluded (#) (see Methods section). In addition, the regression line of leptin on BMI SDS in the whole group of girls (*n*=41) is inserted.

Fasting serum levels of leptin, IGFBP1, insulin, glucose, IGF1, and adiponectin as well as the calculated HOMA-IR are shown in Table 3. Leptin levels correlated with BMI SDS in all boys (*r*=0.79, *P*<0.001), all girls (*r*=0.84, *P*<0.001), and in each separate group (Fig. 2a). A rise of +1 s.d. in BMI SDS increased leptin levels ∼2.3-fold in girls and twofold in boys (Fig. 2a). Girls in general had higher leptin levels than boys and the regression line for leptin levels on BMI SDS in girls was significantly above that in boys (86%, *P*<0.001) (Fig. 2a). A tendency to lower leptin levels in term SGA girls compared with controls was detected (Table 3). However, when leptin levels in relation to BMI SDS were compared between girls' groups, term SGA girls had elevated leptin compared with the combined group of preterm and control girls (deviation 45%, *P*=0.042) (Fig. 2a). Leptin and insulin levels were positively correlated in all boys (*r*=0.62, *P*<0.001) and all girls (*r*=0.63, *P*<0.001). In forward stepwise multiple regression analyses with leptin as dependent variable, 75% of the leptin variability was explained by BMI SDS, insulin, and sex. Replacing insulin by HOMA-IR or addition of height SDS catch-up growth did not increase adjusted *R* ^2^.

#### Leptin levels in adult women

No difference in mean leptin values was observed between the three groups of women. After adjustment for BMI SDS, the leptin level was higher in term SGA than in controls (*P*=0.043) (Table 3). A positive correlation was found between leptin levels and BMI SDS in all women (*r*=0.49, *P*<0.001, *n*=44), with close correlation in preterm AGA women (*r*=0.81, *P*<0.005) in which the slope of the regression line was similar to that in preterm girls. A significant correlation between these two variables was also observed in term SGA women (*r*=0.52, *P*=0.028) (Fig. 2b) but not in controls (*r*=0.42, *P*=0.106). The slope of the regression lines in these two groups was flat, and for a doubling of leptin values, 3 BMI SDS was required (Fig. 2b). The regression lines of leptin on BMI SDS in term SGA women was 53% elevated above that of control women (*P*=0.03). In multiple regression analyses, including all women, only 40% of the leptin variability was explained by BMI SDS, insulin, and group difference (*P*<0.001, *n*=44).

### IGF-binding protein 1

#### IGFBP1 in children

**Figure 3:**
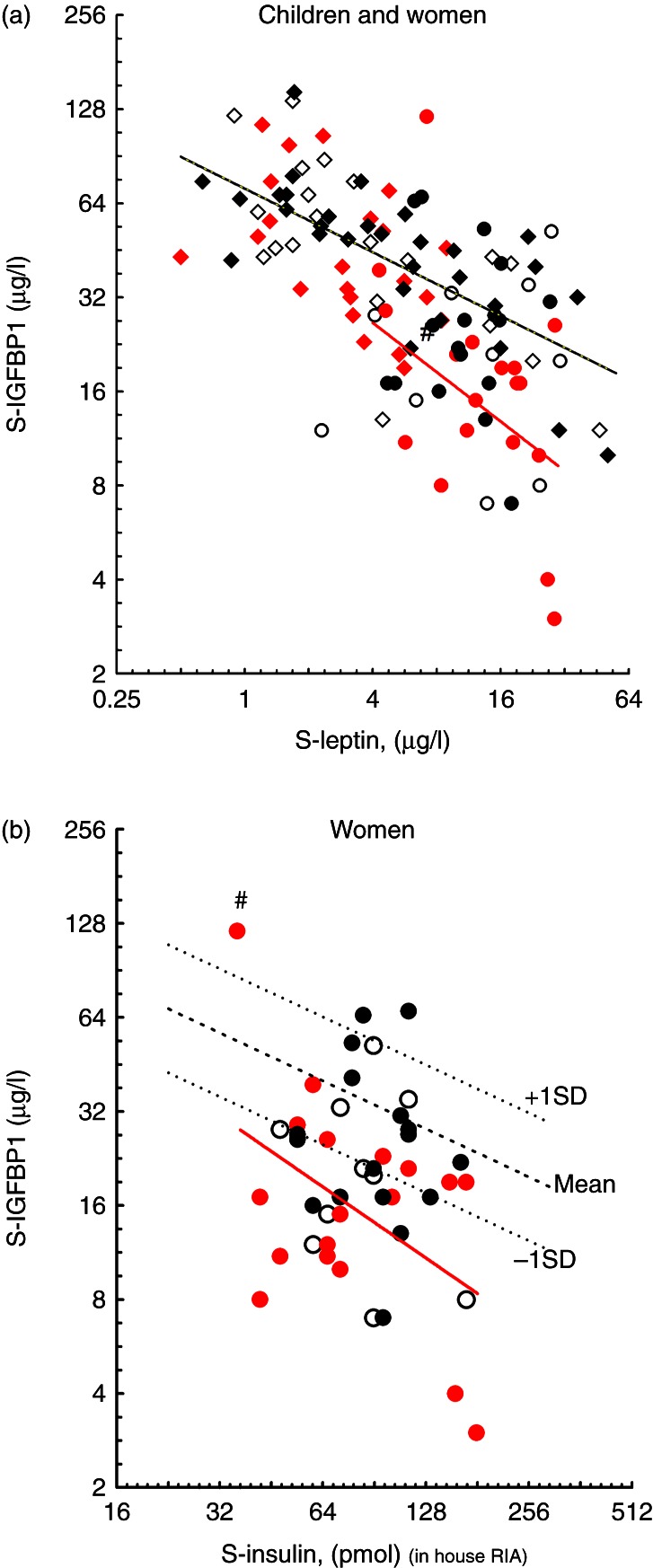
(a) IGFBP1 levels in relation to leptin in children and women. The symbols are the same as in Fig. 1. The black solid line represents the regression line for IGFBP1 on leptin in all children (^2^log *y*=6.1556−0.338*^2^log *x*; *r*=−0.66; *P*<0.001) and the control women (closed circle) did not deviate significantly from this line. The majority of women in the SGA term group (closed circle) were below the regression line for children. The red solid line represents the regression line of IGFBP1 on leptin in term SGA women (closed circle): ^2^log *y*=6.553*−0.694^2^log *x*; *r*=−0.52; *P*=0.028). (b) IGFBP1 in relation to insulin in women. The symbols represent the different groups (open circle, preterm AGA, closed red circle, term SGA, closed black circle, control). The red solid line represents the regression line of IGFBP1 on insulin in all term SGA women (^2^log *y*=7.9778−0.7559* ^2^ log *x*, *r*=−0.46, *P*=0.052). This regression line was about 1.5 s.d. below the mean regression line of IGFBP1 on insulin in a reference group Swedish women with normal OGTT (20), where IGFBP1 and insulin were determined with identical assays. The black dotted and solid lines SDS represent mean and ±1 s.d. of this reference material. Apart from one outlier (#), the term SGA women were below the mean reference line, whereas the control did not differ.

The IGFBP1 levels did not differ between the groups. There was an inverse relationship between IGFBP1 and insulin in children (all children: *r*=−0.71, *P*<0.001; girls: *r*=−0.70, *P*<0.001; and boys: *r*=−0.70, *P*<0.001). IGFBP1 levels were also inversely correlated with leptin in children (all children: *r*=−0.62, *P*<0.001; girls: *r*=−0.68, *P*<0.001; and boys: *r*=−0.52, *P*=0.002) (Fig. 3a). In boys and girls, the regression lines for IGFBP1 on insulin and IGFBP1 on leptin did not differ in the three groups (data not shown). In forward stepwise regression analyses with IGFBP1 as dependent variable in all children, insulin and leptin, but not sex, were shown to be significant independent variables. More than half the IGFBP1 variability (52%) was explained by leptin and insulin (*P*<0.001).

#### IGFBP1 in women

In women, the IGFBP1 level was significantly lower (*P*=0.012) in the term SGA than in controls (Table 3). Term SGA women had higher leptin and lower IGFBP1 than term SGA girls and greater differences of leptin and IGFBP1 were found between the term SGA women and the other women compared to those in the girls (Table 3) (Fig. 3a). IGFBP1 and leptin levels were inversely correlated in term SGA women (*r*=−0.52, *P*=0.028) and the regression line was depressed compared with the other women groups (Fig. 3a).

In term SGA women, IGFBP1 and insulin showed a tendency to inverse correlation (*r*=−0.46, *P*=0.052) but no correlation was observed between IGFBP1 and insulin in the preterm AGA and control women (Table 3 and Fig. 3b). The regression line for IGFBP1 on insulin in the term SGA women was suppressed below the reference material by −1 s.d., a pattern previously noticed in women who later developed diabetes (Fig. 3b) (21).

**Figure 4:**
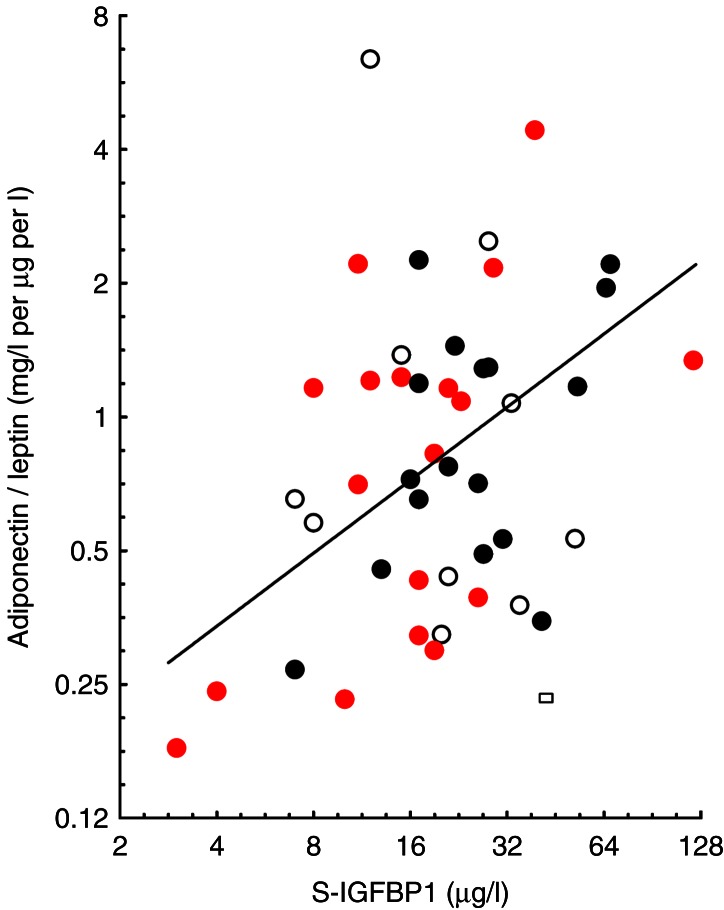
The adiponectin/leptin ratio in relation to IGFBP1 level in women. Symbols as in previous figure (Fig. 3b). The regression lines of the ratio total adiponectin/leptin on IGFBP1 in term SGA subjects (closed circle) (^2^log *y*=−2.7438+0.5856*^2^log *x*; *r*=0.54, *P*=0.022) and in controls (closed circle) (^2^log *y*=−2.6825+0.5357*^2^log *x*, *r*=0.49, *P*=0.045) were superimposable. The black solid line represents their common regression line of term SGA and controls (*r*=0.52; *P*=0.001). The regression line for the preterm AGA group was not significant (*r*= –0.07, *P*= 0.86, line not shown).

A positive association between IGFBP1 and total adiponectin was found in term SGA women (*r*=0.55, *P*=0.021) and controls (*r*=0.57, *P*=0.018) but not in the preterm AGA women. An inverse correlation between total adiponectin and leptin (*r*=−0.60, *P*=0.008) was only observed in term SGA women. In SGA women and controls, the regression lines for the ratio total adiponectin/leptin and IGFBP1 were superimposed with a common correlation coefficient of 0.52 (*P*=0.001) (Fig. 4).

## Discussion

Our novel findings are closer positive correlation between leptin levels and BMI SDS in children than in women and elevated leptin levels in relation to BMI SDS in girls and women born SGA compared with the age-matched control and preterm groups that did not differ from each other. In addition, IGFBP1 levels were inversely correlated with both leptin and insulin in all groups of children as well as in the women born SGA, in which low IGFBP1 was previously reported (23). Similar to women, who develop diabetes, the regression line of IGFBP1 on insulin in women born SGA was suppressed below that in women with normal OGTT over a decade (21).

The obvious disadvantage in this study is the restricted number of subjects in each group, and as our adult subjects are limited to women, we cannot tell whether our findings are applicable to both sexes. Another problem is the elevated BMI SDS in the control groups even if they were properly selected at birth. To compare the data in these two cohorts, we used the same reference to standardize anthropometrical data.

Our girls born SGA remained thin until puberty, a pattern observed in some but not all previous studies (5). In our group of women born SGA, a third had BMI SDS above 1 s.d. but we do not know when they increased in BMI. The women born SGA had LBW SDS than girls born SGA, otherwise they did not differ in anthropometrical characteristics from girls born SGA. Leptin, derived from adipocytes, is considered to reflect the percentage of adipose tissue (12). BMI is commonly used to define obesity in adults but has been considered a poor marker of adiposity during childhood and early stage of puberty, partly due to that BMI does not fully adjust for height in children (29). Therefore, our finding of close association between leptin levels and BMI in the different groups of children was unexpected. In children, a doubling of leptin level was associated with the increase of 1 BMI SDS, whereas 3 BMI SDS was required in women born at term, both in SGA and controls. It is well known that female adults have higher leptin levels than men (30). Higher leptin levels in girls than in boys have previously been reported at the age of 5–10 years in children born SGA and AGA (19).

In our study, the leptin levels after adjustment for BMI SDS were higher in girls born SGA compared with the other girls. In a study of short girls with LBWs and elevated mean BMI SDS, mean leptin level (18 μg/l) was fivefold higher than the mean in our lean SGA girls at the onset of puberty (31). No significant association between leptin and catch-up growth was observed in our study in contrast to some earlier studies, in which higher leptin levels were found in SGA subjects with greater catch-up growth (5, 19, 32). Whether this difference in findings can be attributed to our use of BMI-adjusted values remains unclear for us.

It does not seem likely that the elevated leptin values found in those of our SGA women, who were thin (with BMI SDS below −1 s.d.), can be attributed to leptin resistance. We instead propose that the increase in leptin levels could be a compensatory event in subjects born SGA to enhance hepatic insulin sensitivity. Leptin can partly replace the absence of adipose tissue in lipodystrophic patients, who develop insulin-resistant diabetes and elevated triglycerides. In an ongoing study at NIH of 55 patients with generalized and partial lipodystrophy, leptin therapy reduced both HBA1c and triglyceride levels (33). In rodents with diabetes, leptin treatment normalized plasma glucose and increased insulin sensitivity (34). Apart from reducing the glucose output, an increase in hepatic insulin sensitivity is expected to inhibit IGFBP1 production leading to greater availability of IGFs at their target tissues.

Peripheral insulin resistance in subjects born SGA at term has been described in both children and adults (5, 6), and in our children born SGA at term, we have recently reported elevated insulin and HOMA-IR values and a tendency to lower IGFBP1 after BMI adjustment (7). The inverse relationship between IGFBP1 and insulin is well established and IGFBP1 has been used as a surrogate marker of hepatic glucose output (20). In this study, we show that the IGFBP1 levels in children were inversely related to both insulin and leptin. These two variables together explained more than half of the IGFBP1 variability (53%) in the whole group of children, which could support our hypothesis that leptin enhances hepatic insulin sensitivity. Inverse association between IGFBP1 and leptin has previously been reported in children born SGA by Challa *et al*. (5). In middle-aged women, IGFBP1 levels rose after the onset of diabetes, indicating a loss of insulin sensitivity in the liver (21). In this context, it is of interest that the IGFBP levels in SGA and control women were inversely related to the ratio total adiponectin/leptin. This ratio is considered to be a better predictor for diabetes than total adiponectin alone (35).

In summary, we found elevated leptin levels in relation to BMI in girls and women born SGA and lower IGFBP1 levels in women born SGA. IGFBP1 levels were inversely correlated with leptin in all children and in the women born SGA. IGFBP1 levels in relation to insulin were suppressed in women born SGA. In children, leptin and insulin were strong suppressors of IGFBP1 levels. We speculate that the higher leptin levels could be a protective event to enhance hepatic insulin sensitivity. In conclusion, the impact of intrauterine events leading to fetal starvation appears to be a risk factor for metabolic changes later in life.

## Author contribution statement

A Kistner and M Vanpée participated in study design, collection, analysis, and interpretation of the data as well as writing the manuscript. K Hall took part in analysis, interpretation, writing, and reviewing the manuscript.

## Figures and Tables

**Table 1:** Birth characteristics for the children and adult women and maternal characteristics for the three groups. Values represent medians (ranges).

	**Groups**			***P* values**			
	1. Preterm AGA	2. Term SGA	3. Control	ANOVA	1 vs 3	2 vs 3	1 vs 2
GA at birth (weeks)							
Boys (*n*=13, 10, and 12)	26 (24 to 30)	40 (38 to 40)	40 (38 to 41)	<0.001*	<0.001*	0.28	<0.001*
Girls (*n*=9, 13, and 19)	27 (24 to 29)	40 (37 to 41)	40 (38 to 42)	<0.001*	<0.001*	0.90	<0.001*
Women (*n*=10, 18, and 17)	30 (28 to 32)^†^	40 (37.5 to 42)	40.5 (39 to 42)^†^	<0.001*	<0.001*	0.29	<0.001*
BW (g)							
Boys	960 (720 to 1410)	2430 (2090 to 2780)	3570 (3020 to 4760)	<0.001*	<0.001*	<0.001*	<0.001*
Girls	890 (610 to 1260)	2640 (1780 to 2860)	3400 (2700 to 4470)	<0.001*	<0.001*	<0.001*	<0.001*
Women	1340 (990 to 2040)	2115 (1610 to 2660)	3650 (3120 to 4220)	<0.001*	<0.001*	<0.001*	<0.001*
BW SDS							
Boys	−0.87 (−1.78 to 0.93)	−3.11 (−3.90 to −2.62)	−0.28 (−1.94 to 1.37)	<0.001*	0.27	<0.001*	<0.001*
Girls	−0.94 (−1.11 to 0.19)	−2.46 (−4.18 to −2.14)	−0.34 (−1.53 to 1.62)	<0.001*	0.08	<0.001*	<0.001*
Women	−1.50 (−1.95 to 0.05)	−4.13 (−6.21 to −2.89)^†^	−0.07 (−1.75 to 1.15)	<0.001*	<0.001*	<0.001*	<0.001*
Birth length SDS							
Boys	−0.56 (−3.93 to 3.40)	−2.81 (−4.05 to −1.27)	−0.10 (−2.01 to 1.17)	<0.001*	0.75	<0.001*	<0.001*
Girls	−1.45 (−3.67 to 0.76)	−2.08 (−5.32 to −0.14)	−0.91 (−3.01 to 1.80)	<0.001*	0.36	<0.001*	0.017*
Women	−0.50 (−3.29 to 0.85)	−3.68 (−5.76 to −1.92)	−0.14 (−2.57 to 1.80)	<0.001*	0.42	<0.001*	<0.001*
Maternal age (years)							
Boys	30 (23 to 38)	27 (22 to 40)	29 (23 to 40)	0.78	0.94	0.57	0.52
Girls	28 (23 to 37)	29 (22 to 36)	30 (21 to 37)	0.52	0.48	0.27	0.79
Women	24 (17 to 35)*	25 (17 to 42)	25 (21 to 41)	0.17	0.06	0.36	0.25
Maternal height SDS							
Boys	−0.43 (−2.24 to 0.89)	−0.75 (−1.91 to 0.40)	−0.42 (−2.07 to 1.22)	0.59	0.97	0.36	0.38
Girls	−0.76 (−2.56 to 1.22)	−0.75 (−2.40 to 0.56)	0.07 (−1.74 to 1.71)	0.42	0.44	0.20	0.71
Women		−1.71 (−2.07 to 1.22)					
Maternal pre-pregnancy weight (kg)							
Boys	58 (50 to 80)	59 (50 to 75)	60 (53 to 72)	0.91	0.94	0.84	0.90
Girls	60 (49 to 108)	59 (49 to 80)	62 (45 to 78)	0.91	0.77	0.85	0.66
Women	62 (57 to 68)	55 (43 to 68)	57 (50 to 72)	0.039*	0.21	0.12	0.014
Maternal pregnancy weight gain (kg)							
Boys	6 (2 to 13)	13 (7 to 23)	15 (8 to 35)	0.001	<0.001	0.16	0.01
Girls	10 (4 to 18)	11 (4 to 16)	13 (6 to 27)	0.05	0.043	0.044	0.84
Women	10 (8 to 20)	10 (5 to 21)	13 (−13 to 20)	0.89	0.66	0.73	0.85
Maternal pre-pregnancy BMI (kg/m^2^)							
Boys	21 (19 to 27)	22 (19 to 28)	22 (19 to 25)	0.90	0.88	0.66	0.77
Girls	21 (19 to 36)	22 (19 to 29)	22 (18 to 32)	0.85	0.59	0.71	0.85
Women		21 (17 to 28)					

*P values <0.05 according to ANOVA followed by *post hoc* Fisher's test between groups. ^†^ *P* value (<0.01) according to ANOVA (*t*-test) for the indicated group (women) vs corresponding girl group. GA, gestational age; BW, birth weight.

**Table 2:** Age, height, and BMI for the boys, girls, and women in the three groups at the time of the study and target height for the children (see Methods section). Values represent medians (ranges).

	**Groups**			***P* values**			
	1. Preterm AGA	2. Term SGA	3. Control	ANOVA	1 vs 3	2 vs 3	1 vs 2
Age (years)							
Boys	9.4 (9.1 to 10.0)	9.9 (9.4 to 10.0)	9.9 (9.8 to 10.0)	<0.001*	<0.001*	0.25	<0.001*
Girls	9.5 (9.2 to 10.0)	10 (9.1 to 10.0)	9.9 (9.4 to 10.0)	0.06	0.033*	0.81	0.029*
Women	23 (19 to 29)^†^	25 (24 to 29)^†^	25 (24 to 30)^†^	0.028	0.09	0.23	<0.01*
Weight							
Boys	29 (24 to 44)	30 (25 to 37)	36 (23 to 49)	0.06	0.06	0.029*	0.64
Girls	29 (23 to 47)	28 (19 to 36)	36 (27 to 61)	0.031*	0.17	0.01*	0.35
Women	64 (50 to 80)^†^	57 (45 to 77)^†^	65 (55 to 89)^†^	0.006*	0.38	<0.01*	0.06
Weight SDS							
Boys	−0.62 (−2.47 to 2.80)	−0.40 (−1.99 to 1.02)	1.16 (−0.70 to 3.26)	0.16	0.22	0.06	0.43
Girls	−0.84 (−2.30 to 2.85)	−1.07 (−3.73 to 0.85)	0.63 (−1.35 to 3.24)	0.041	0.28	0.012*	0.25
Weight SDS difference (birth to current age)							
Boys	0.54 (−2.21 to 3.78)	2.52 (1.15 to 4.87)	0.97 (−1.56 to 3.08)	0.020*	0.56	0.007*	0.030*
Girls	0.47 (−1.26 to 3.43)	1.92 (−0.20 to 4.50)	0.55 (−2.64 to 3.27)	0.030*	0.88	0.016*	0.031*
Height (cm)							
Boys	136 (123 to 144)	136 (131 to 140)	145 (126 to 156)	0.004*	<0.01*	<0.01*	0.98
Girls	137 (121 to 150)	136 (122 to 147)	140 (130 to 161)	0.11	0.057*	0.57	0.13
Women	168 (152 to 180)^†^	160 (149 to 169)^†^	168 (156 to 179)^†^	0.001*	0.48	<0.001*	0.016*
Height SDS							
Boys	−0.46 (−2.64 to 0.98)	−0.72 (−1.73 to −0.11)	0.80 (−0.98 to 2.44)	0.017*	0.023*	<0.01*	0.57
Girls	−0.73 (−2.85 to 2.24)	−0.83 (−2.91 to 1.35)	−0.14 (−1.44 to 1.84)	0.21	0.14	0.15	0.86
Women	−0.02 (−2.56 to 2.04)	−1.25 (−3.06 to 0.23)	0.769 (−0.94 to 3.68)	0.001*	0.47	<0.001*	0.018*
Height SDS difference (birth to current age)							
Boys	−0.08 (−3.78 to 2.92)	1.89 (0.0 to 3.34)	1.04 (−0.54 to 2.60)	0.005*	0.041*	0.15	0.001*
Girls	0.47 (−1.26 to 3.43)	2.01 (−0.83 to 4.47)	0.93 (−1.70 to 3.50)	0.035*	0.74	0.028*	0.022*
Women	1.04 (−1.88 to 2.36)	2.69 (−0.31 to 5.01)	0.66 (−1.65 to 2.98)	<0.001*	0.92	<0.001*	<0.001*
BMI							
Boys	16.4 (14.9 to 21.4)	16.6 (14.6 to 19.7)	17.3 (14.3 to 22.5)	0.67	0.74	0.38	0.56
Girls	16.4 (14.3 to 24.8)	15.4 (13 to 18.3)	18.4 (14.2 to 23.9)	0.016*	0.07	0.005*	0.49
Women	22.8 (18.6 to 29.0)^†^	20.5 (16.2 to 27.8)^†^	23.9 (19.3 to 31.7)^†^	0.36	0.63	0.16	0.46
BMI SDS							
Boys	0.08 (−1.22 to 2.25)	0.08 (−1.42 to 1.55)	0.36 (−1.23 to 2.50)	0.73	0.97	0.49	0.51
Girls	0.06 (−1.41 to 2.68)	−0.78 (−2.67 to 0.76)	0.83 (−1.78 to 2.25)	0.009*	0.58	0.003*	0.038*
Women	0.35 (−1.20 to 2.66)	−0.44 (−2.07 to 2.23)	0.77 (−0.94 to 3.68)	0.40	0.61	0.18	0.52
Target height SDS							
Boys	−0.14 (−1.28 to 1.15)	−0.56 (−1.21 to 0.62)	−0.29 (−2.12 to 1.23)	0.51	0.98	0.33	0.30
Girls	−0.10 (−1.17 to 1.14)	−0.14 (−1.66 to 0.23)	−0.10 (−1.99 to 1.88)	0.32	0.81	0.14	0.31

*P values <0.05 according to ANOVA followed by the *post hoc* Fisher's test between groups. ^†^ *P* values <0.01 according to ANOVA (*t*-test) for the indicated group (women) vs corresponding girl group.

**Table 3:** Fasting levels of leptin, IGFBP1 glucose, insulin, and IGF1 in the three groups of children and in the adult subjects as well as adiponectin in the adult subjects. Values of HOMA-IR are also shown. Values are presented as geometrical mean (±95% CI) except for glucose, which is presented as mean (95% CI).

	**Groups**			***P* values**			
	1. Preterm AGA	2. Term SGA	3. Control	ANOVA	1 vs 3	2 vs 3	1 vs 2
Leptin (μg/l)							
Boys	3.8 (2.0–7.0)	2.5 (1.2–5.0)	3.0 (1.6–5.7)	0.64	0.59	0.68	0.35
Girls	5.3 (2.7–10.2)	3.4 (2.0–5.9)	6.5 (4.1–10.2)	0.20	0.60	0.08	0.31
Women	11.8 (7.9–17.6)	13.0 (9.7–17.6)^†^	10.2 (7.4–13.9)	0.51	0.56	0.25	0.68
IGFBP1 (μg/l)							
Boys	47 (34–66)	46 (32–67)	51 (37–72)	0.90	0.73	0.67	0.92
Girls	42 (28–61)	43 (32–60)	40 (30–52)	0.91	0.83	0.67	0.87
Women	19 (12–30)	14 (10–20)^†^	25 (18–35)	0.041*	0.32	0.012*	0.21
Glucose (mmol/l)							
Boys	4.4 (4.1–4.8)	4.6 (4.2–5.0)	4.1 (3.7–4.5)	0.15	0.19	0.06	0.48
Girls	4.3 (4.0–4.6)	4.4 (4.1–4.7)	4.1 (3.9–4.4)	0.33	0.38	0.15	0.69
Women	4.4 (4.1–4.7)	4.3 (4.1–4.5)	4.3 (4.1–4.4)	0.65	0.44	0.92	0.37
Insulin (pmol/l)							
Boys	25 (18–36)	29 (20–43)	21 (18–36)	0.81	0.98	0.56	0.58
Girls	26 (18–38)	35 (26–48)	32 (24–42)	0.46	0.38	0.64	0.22
Women (corrected)	42 (32–54)	39 (32–47)	45 (37–55)	0.55	0.59	0.28	0.69
HOMA-IR							
Boys	5.0 (3.4–7.4)	6.0 (3.9–9.2)	4.5 (3.1–6.7)	0.61	0.72	0.33	0.52
Girls	4.9 (3.3–7.2)	6.8 (4.9–9.4)	5.8 (4.5–7.7)	0.42	0.46	0.48	0.19
Women	8.1 (6.0–10.9)	7.4 (5.6–9.8)	8.6 (7.3–10.1)	0.61	0.74	0.32	0.60
IGF1 (μg/l)							
Boys	192 (157–235)	181 (145–225)	153 (125–187)	0.25	0.11	0.26	0.68
Girls	288 (239–347)	201 (172–234)	234 (205–268)	0.017*	0.07	0.14	0.004*
Women	255 (231–281)	205 (179–234)	213 (198–230)	0.029*	0.035*	0.55	0.009*
Adiponectin (mg/l)							
Women	10.3 (8.0–13.2)	9.8 (8.1–11.8)	9.4 (7.7–11.4)	0.84	0.56	0.74	0.77
Leptin (μg/l), ANCOVA, adjusted BMI SDS							
Women	11.8 (8.4–16.5)	14.3 (11.1–18.4)	9.2 (7.0–12.0)	0.07	0.49	0.043**	0.71

*P values <0.05 according to ANOVA followed by the *post hoc* Fisher's test between groups; ** *P* value <0.05 according to *post hoc* planned comparison and Bonferroni correction by analysis of covariance (ANCOVA); ^†^ *P* values <0.01 according to ANOVA (*t*-test) for the indicated group (women) vs corresponding girl group.
